# First case of childhood Takayasu arteritis from Syria: a case report

**DOI:** 10.1186/s13256-021-03077-w

**Published:** 2021-09-23

**Authors:** Wafa Alwattar, Rawan Al khudari, Judy Naameh, Jia Batha, Raghad Almajzoub, Basheer Khalil

**Affiliations:** 1 Department of Gynaecology and Obstetrics, Faculty of Medicine, Damascus University, Damascus, Syria; 2 Department of Paediatrics, Children’s University Hospital, Faculty of Medicine, Damascus University, Damascus, Syria; 3 Department of Internal Medicine, Faculty of Medicine, Damascus University, Damascus, Syria; 4 Department of Gastrology, Faculty of Medicine, Damascus University, Damascus, Syria; 5 Division of Rheumatology, Department of Paediatrics, Children’s University Hospital, Faculty of Medicine, Damascus University, Damascus, Syria

**Keywords:** Takayasu arteritis, Childhood, Pulselessness, Arabs, Syria

## Abstract

**Background:**

Takayasu arteritis is a systemic granulomatous inflammation affecting the large- and medium-sized vessels such as aorta, its main branches, and pulmonary and renal arteries. Childhood Takayasu arteritis is a subtype of Takayasu arteritis that affects the age group ranging from young infants to late adolescents.

**Case presentation:**

We report the first childhood Takayasu arteritis case from Syria, a 12-year-old Syrian girl presenting with nonspecific symptoms and signs plus ischemic clinical features in her left arm. She relapsed twice with different additional symptoms each time.

**Conclusions:**

There is scarcity of reviews and studies on childhood Takayasu arteritis in Arabs. We aim to share our experience to keep childhood Takayasu arteritis in mind as a differential diagnosis in any child presenting with hypertension, absent or reduced peripheral arterial pulse, or blood pressure differences between extremities.

## Background

Takayasu arteritis (TA) is a systemic granulomatous inflammation affecting the large- and medium-sized vessels such as aorta, its main branches, and pulmonary and renal arteries. The inflammation and infiltration of plasma cells, giant cells, and lymphocytes lead to stenosis, occlusion, dilatation, and aneurysms of the vessels [1].

It occurs in female patients more than in males and is most prevalent in Japan, South East Asia, India, and Mexico [2]. Although age of onset ranges from 10 to 40 years [1], the highest incidence occurs during the third decade of life [3]. However, childhood TA (c-TA), which is a subtype of TA, affects the age group ranging from young infants to late adolescents [4].

Furthermore, the incidence of TA worldwide has been estimated to be 2/1,000,000 per year. However, the prevalence and incidence of TA in children is unknown [5]. We could not find any other registered cases of c-TA in Children’s University Hospital in Damascus, which is the biggest center for treating children in Syria, from 2000 till now; thus, we report the first known case from Syria.

Clinical manifestations involve two phases: an early phase, “pre-pulseless phase,” with nonspecific systemic inflammatory symptoms such as weight loss, fever, night sweats, asthenia, headache, arthralgia, and muscle pain; the disease is not often recognized in this phase, and if the disease is untreated, a “pulseless phase” with multiple arterial occlusions and stenosis will occur, causing acute ischemic symptoms like seizures, congestive heart failure, and high blood pressure [5].

Diagnosis of childhood TA is difficult and challenging because of its nonspecific symptoms that delay the diagnosis and the treatment [5].

Treatment of childhood TA is glucocorticoids and immunosuppressants [6]. Stents, bypass grafts, and angioplasty may be used in irreversible arterial stenosis cases [7].

We report the first case in a child from Syria who presented with nonspecific and ischemic symptoms in her left arm. Conventional angiography showed narrowing in the inlet of the left subclavian artery extending to the branching point of the left vertebral artery. Later, she relapsed twice with different additional symptoms each time.

## Case presentation

A 12-year-old Syrian girl was admitted to Children's University Hospital of Damascus due to 3 months complaints of a variety of unexplained symptoms. She had generalized myalgia and arthralgia without inflammatory signs. The pain was most severe in her left shoulder and elbow, with numbness and tingling in her left arm. She also had exertional dyspnea, pallor, dizziness, and constitutional symptoms (fatigue, fever with night sweats, anorexia, and loss of approximately 7% of her weight), along with intermittent epigastric abdominal pain that exacerbated with meals and was occasionally associated with vomiting and diarrhea. In addition, she developed headache and episodes of epistaxis during her admission.

Her past medical history was significant for two episodes of tonsillitis during the last year, and she was diagnosed with iron deficiency anemia in an outpatient clinic. She was treated with penicillin G benzathine (dose: 1.2 million units intramuscular injection every 2 weeks), and iron was replaced without any improvement.

Clinical examination revealed lymphadenopathy in the cervical (1 × 1 cm) and inguinal (1 × 2 cm) regions. Her pulse was absent in the left brachial and radial arteries, and blood pressure was unobtainable in her left arm. Her blood pressure (BP) was 130/70 and 140/90 mmHg in her right arm and lower limbs respectively. Vascular bruits were heard over the left supra- and subclavian areas on auscultation.

Laboratory tests showed microcytic anemia with elevation in white blood cells (WBCs) count, platelet count, and inflammatory markers, including C-reactive protein (CRP) and erythrocyte sedimentation rate (ESR) (values are presented in Table 1). Tests also revealed mild elevation in anti-streptolysin O (576 U/ml, normal range 50–250 U/ml), iron deficiency (3 μmol/l, normal range 9–31.3 μmol/l), elevated Von Willebrand factor (189%, normal range 60–150%). Liver function tests, blood urea nitrogen, creatinine, prothrombin time, and partial thromboplastin time were all normal. The following tests were all within normal limits: Widal test, Wright test, tuberculin skin test, direct and indirect Coombs, cold agglutinins, rheumatoid factor, anti-DNA, antinuclear antibody, perinuclear anti-neutrophil cytoplasmic antibodies, cytoplasmic antineutrophil cytoplasmic antibodies, complement factors C3 and C4, cytomegalovirus and Epstein–Barr virus antibodies, and anti-hepatitis A virus IgM antibodies.

**Table 1:** Patient’s course of the disease

	Clinical findings		Blood tests						Imaging	Treatment*
	Symptoms	Significant signs	Hemoglobin (g/dl)	Mean corpuscular volume (fl)	WBCs (cell/μl) (neutrophil/lymphocyte)	ESR (mm/hour) (normal range for females ≤ 20 mm/hour)	Platelets (platelet/μl)	CRP (mg/dl) (normal range 0–10 mg/dl)		
When diagnosed	Generalized myalgia and arthralgia, severe pain in the left shoulder and elbow with numbness and tingling in the left arm, dyspnea, pallor, constitutional symptoms, intermittent epigastric abdominal pain occasionally associated with vomiting and diarrhea, headache, and epistaxis	Lymphadenopathy, pulselessness with unobtainable BP in the left arm. BP was 130/70 and 140/90 mmHg in the right arm and lower limbs, respectively. Bruits over the left supra- and subclavian areas	10	70	13,200 (77/12)	113	731 × 10^3^	35.5	Figs. 1, 2	Prednisolone 1 mg/kg/day Methotrexate 10 mg/m^2^/weak Aspirin 1–2 mg/kg/day
First relapse (after 4 months)	Chest pain radiating to the left arm, syncope, dyspnea, fever, and fatigue	Bruits over the left and right supra- and subclavian areas. Pulselessness with unobtainable BP in both arms. BP in the lower limbs was elevated (150/80 mmHg)	11.5	73	14,800 (74/19)	99	660 × 10^3^	12.7	Fig. 3	As above Cyclophosphamide 1000 mg/m^2^/month Amlodipine 5 mg/day for hypertension
Second relapse (after 8 months)	Occipital headache, abdominal pain, vomiting, arthralgia, and fatigue	Cushing appearance, bruits over the right supra- and subclavian areas, pulselessness with unobtainable BP in both arms. BP in the lower limbs was 138/84 mmHg	10.4	83	15,800 (88/16)	122	466 × 10^3^	15.6	Fig. 4A, B	Prednisolone and cyclophosphamide were continued as described above. Methotrexate was replaced with azathioprine 100 mg/day. Amlodipine 5 mg/day Aspirin 1–2 mg/kg/day
Last follow-up (after 14 months)	Only mild arthralgia at the knees, all other symptoms were relieved	Pulse was present in four extremities. BP was measurable in four extremities and was approximately 120/70 mmHg	13.4	84	8300 (77/15)	5	384 × 10^3^	20	Fig. 5A, B	Prednisolone (1 mg/kg) Azathioprine 2 mg/kg/day Amlodipine 5 mg/day Aspirin 1–2 mg/kg/day

*When diagnosed and in each relapse; treatment was initiated with intravenous methylprednisolone 30 mg/kg/day for 3 days

Bone marrow aspiration and biopsy showed no significant abnormalities. Chest X-ray and pharyngeal swap were also normal.

**Fig. 1:**
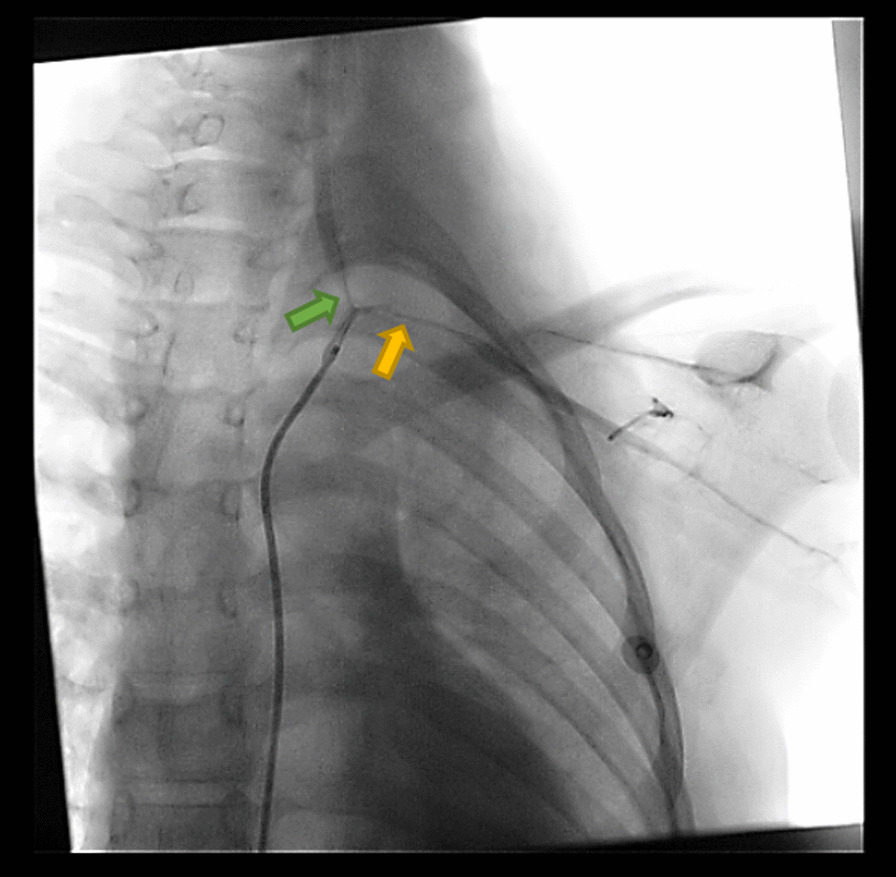
Conventional angiography showing narrowing of the left subclavian artery (yellow arrow) extended to the branching point of the left vertebral artery (green arrow) and followed by widening of the left subclavian artery

**Fig. 2:**
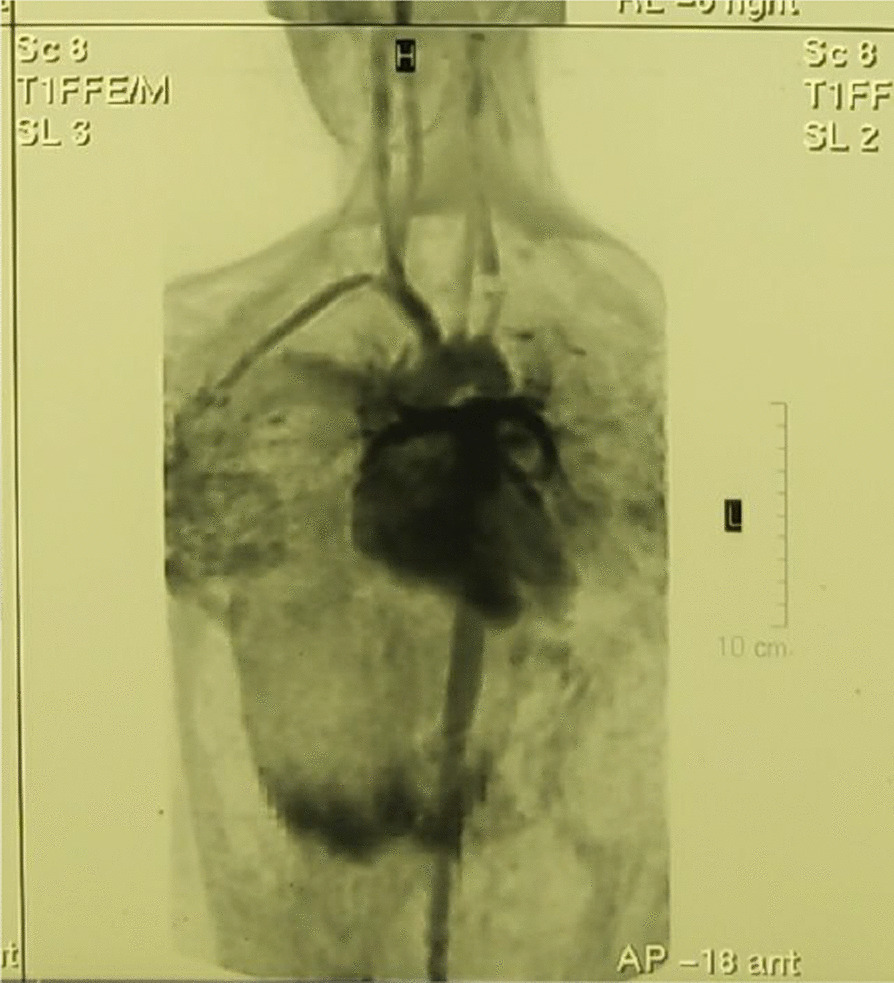
Magnetic resonance angiogram showing injury of the left subclavian artery

Conventional angiography was performed regarding the difference in pulse and blood pressure values between the two upper limbs. It showed narrowing in the inlet of the left subclavian artery extending to the branching point of the left vertebral artery, and this was followed by widening of the left subclavian artery (Fig. 1). Other arteries were normal. The results were supported with magnetic resonance angiography (MRA, Fig. 2), and according to these results the diagnosis of childhood Takayasu arteritis was established.

She was given 30 mg/kg of intravenous methylprednisolone daily for 3 days as an initial management, then she was treated conservatively with close follow-up. Her treatment included prednisolone 1 mg/kg/day and methotrexate 10 mg/m^2^/week for 3 months along with aspirin 1–2 mg/kg, omeprazole, folic acid, calcium, and vitamin D. The clinical features of ischemia (that is, pulselessness), BP, complete blood count, ESR, and CRP were used for clinical follow-up.

After that, the patient relapsed twice when prednisolone was tapered off to a maintenance dosage, and she had additional symptoms during her relapses (details are presented in Table 1).

**Fig. 3:**
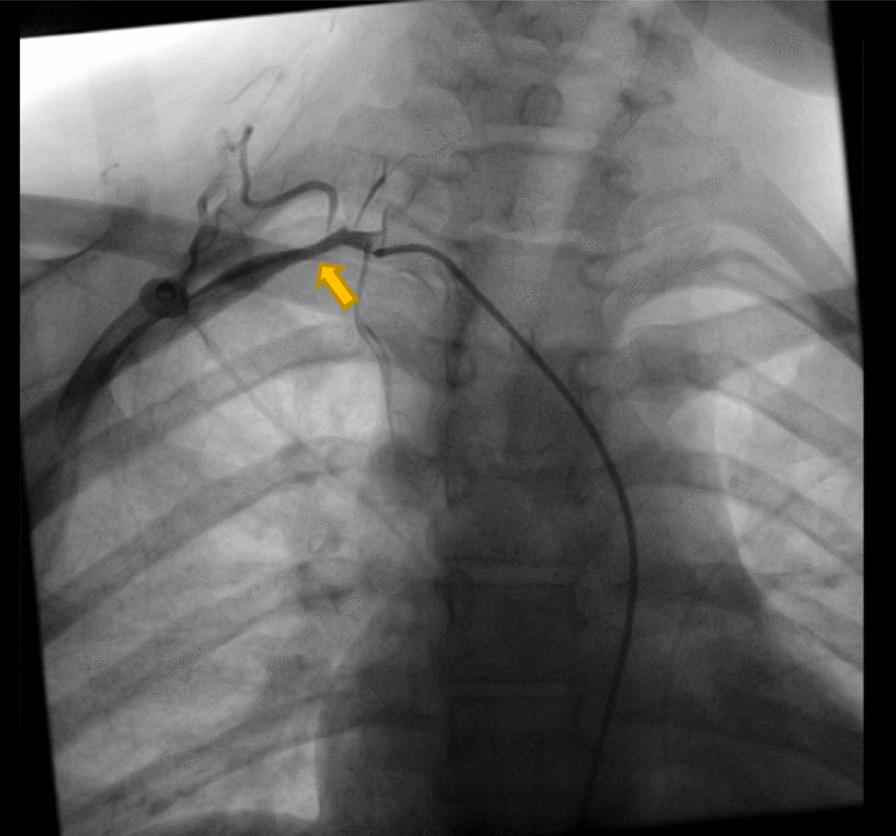
Conventional angiography showing narrowing of the right subclavian artery (yellow arrow)

**Fig. 4:**
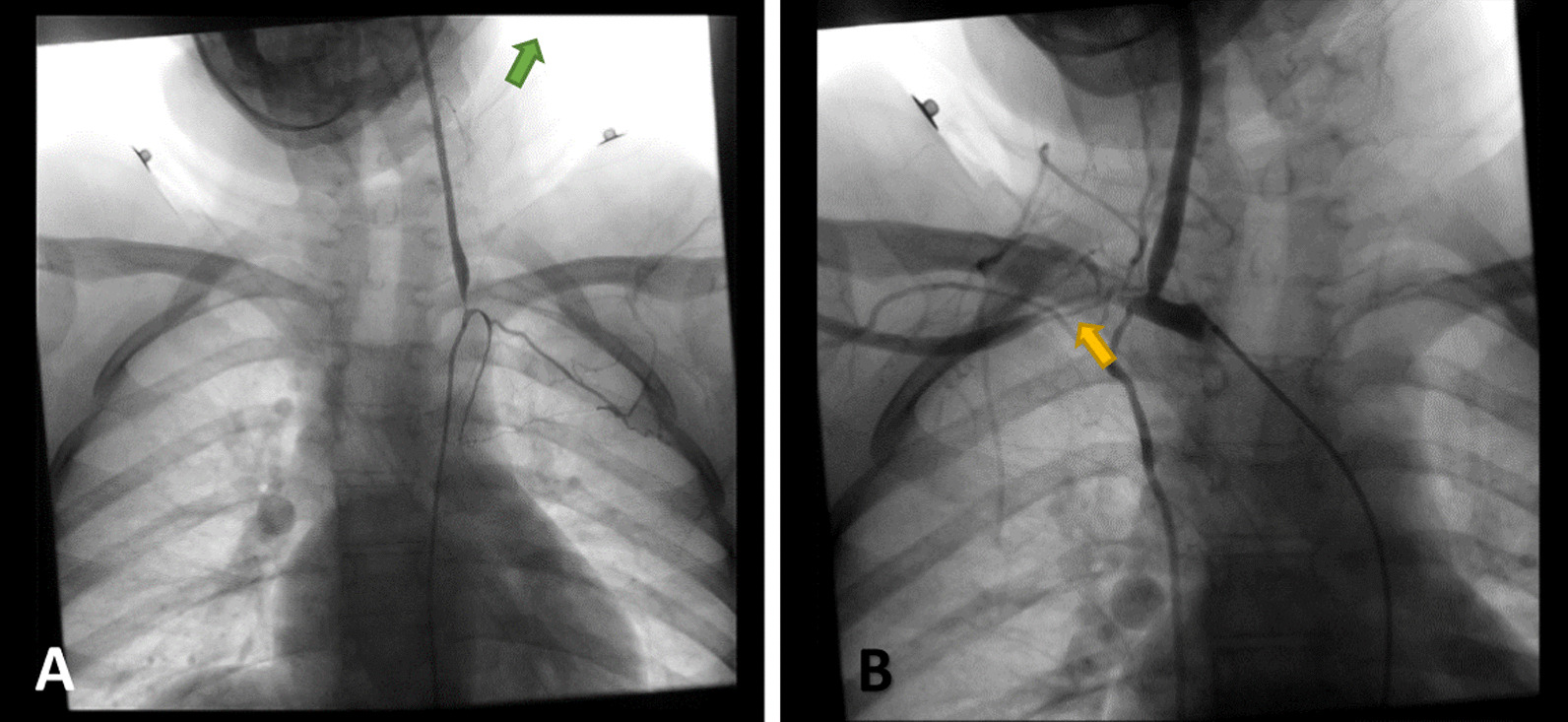
**A**, **B** Conventional angiography. **A** shows complete occlusion of the left subclavian artery, and collateral arteries are visible (green arrow). **B** shows increased narrowing of the right subclavian artery (yellow arrow) compared with Fig. 3

Her first relapse was after 4 months, and the main complaint was chest pain that radiated to her left arm. On admission, a cardiac catheter was performed and no injury in coronary arteries was found. Electrocardiography, creatine kinase, troponin, arterial blood gases, and echocardiography were all normal. The pain was relieved by nitroglycerin patches. Conventional angiography showed that her disease was progressing radiologically (in addition to the narrowing of the left subclavian artery, the right subclavian artery was affected; Fig. 3). Prednisolone was returned to initial dose with methotrexate, and cyclophosphamide 1000 mg/m^2^/month was added. She also had hypertension that was managed with amlodipine 5 mg/day. Despite adding cyclophosphamide, she had a second relapse 4 months later when prednisolone dose was reduced. Her chief complaint was severe occipital headache. The disease was still progressing radiologically (Fig. 4A, B). Therefore, methotrexate was replaced with azathioprine 2 mg/kg/day with continuation of prednisolone and cyclophosphamide.

**Fig. 5:**
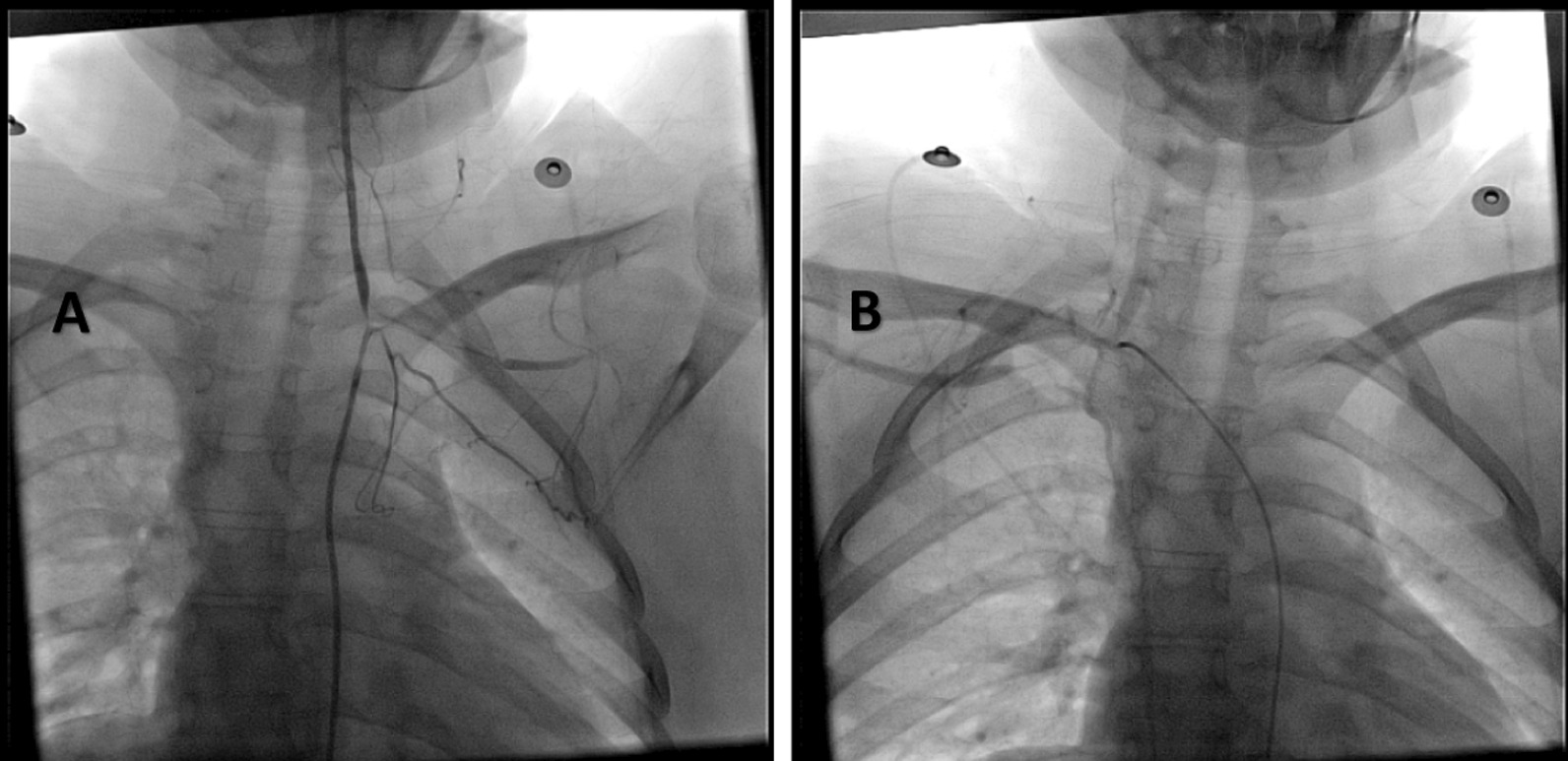
**A**, **B** Conventional angiography. **A** shows only mild improvement in the left subclavian artery, and **B** shows no regression in the injury of the right subclavian artery despite clinical improvement

The patient was followed-up closely, and after approximately 14 months since the onset of the disease; her clinical and laboratorial condition was stable (details are presented in Table 1), although radiological findings did not regress (Fig. 5A, B). The treatment was adjusted to azathioprine 1 mg/kg/day and prednisolone 1 mg/kg/day. The patient is still following up in our outpatient clinic for continuous evaluation and dosage adjustment.

## Discussion

Takayasu arteritis (TA) is a chronic granulomatous inflammation of large- and medium-sized arteries of unknown etiology, affecting mainly aorta and its large branches and causing vessel stenosis, occlusion or dilation, and aneurysmal formation [1]. It can also involve the pulmonary, abdominal, and coronary arteries [3, 5].

TA rarely occurs in children [3]. The exact incidence of childhood Takayasu arteritis (c-TA) is not determined yet [5]. Despite the high incidence of TA in East Asian countries, it is not limited to one race [8, 9]. There is a low prevalence of TA in Arabs [10].

The pattern of involvement of arteries varies among the races [8]. The ascending aorta, aortic arch, and its branches are mostly affected in Japanese patients, while thoracic and abdominal aorta are commonly affected in Indian and North American patients [1, 8]. Of note, there is no similar study on c-TA in Arabs. However, a systematic review of TA in Arabs including 197 patients, with a mean age of 28 years, from seven Arab countries, not including Syria, shows a predominant involvement of the aortic arch branches [10].

We described the first case of childhood Takayasu arteritis (c-TA) reported from Syria. In our case, a 12-year-old girl with TA affecting the aortic branches presented with a group of nonspecific symptoms and signs of this rare disease. TA diagnosis in children is frequently delayed as compared with adults [3, 5]. Generally, delay in diagnosis ranges from 1 month to 5 years [11]. In our case, the girl was diagnosed after 3 months from the beginning of symptoms. TA manifestation differs in children from adults; c-TA probably presents with less specific signs and symptoms [5]. Clinical manifestations in children have a broad spectrum and can be divided into constitutional symptoms, which appear in 50% of patients in early stage of disease, and organ-specific symptoms, which occur later [5, 8]. Our patient presented with both types of symptoms. However, nonspecific manifestations of c-TA make its diagnosis challenging for clinicians [5]. As a result, arterial inflammation progresses to a stage of chronic stenosis and gradual arterial narrowing that causes ischemia of the tissues [3]. Patients often seek medical help in the sequelae stage [3].

Arterial hypertension is the predominant manifestation in both adult and child patients [5, 8]. Other common clinical features in children are headache, fever, dyspnea, weight loss, vomiting, abdominal pain, myalgia, and arthralgia [12]. Our patient had all of these symptoms when diagnosed, and then she relapsed twice, with different manifestation in each one. Our patient also has lymphadenopathy in cervical and inguinal regions, which is an uncommon finding in c-TA [12].

There is yet an unclear association between TA and TB (tuberculosis), as 20% of TA patients were diagnosed with active TB [4, 5]. Nevertheless, our patient did not have TB.

The diagnosis of TA in this case was based on the criteria developed by the Ankara conference in 2008, which showed 100% sensitivity and 99.9% specificity [13].

The criteria include the following standards: one mandatory criterion that angiographic abnormality should be proved by imaging studies, plus one of the following: absent/weak or unequal peripheral arterial pulses, discrepancy of four limb systolic blood pressure measurements of more than 10 mmHg difference in any limb, bruits over large arteries, hypertension, and raised acute phase reactant (ESR < 20 or elevated CRP) [13].

Our patient met every criterion mentioned above when she was first diagnosed except for hypertension, which developed later within the first relapse.

In terms of classification and depending on Numano’s classification, our patient is considered type I, as she showed MRA involvement of the left subclavian artery without any involvement of the ascending aorta or the aortic arch [11].

Our patient presented with clinical features highly suggestive of vascular morbidity; thus, conventional arteriography was performed, followed by MRA, which is considered a sensitive and an accurate tool for establishing the diagnosis and continuing the follow-up of Takayasu arteritis [8].

Treatment of TA depends on glucocorticoids as a first line agent; however, high relapse rate (occurring in 46–84% of patients) is increasingly suggesting the use of second-line immunosuppressants to maintain remission [6].

Our patient was started on both (glucocorticoids and methotrexate); however, she still relapsed upon reduction of steroid dose, and this highlights the challenge of finding a way to assess disease activity and guide successful therapy [5].

Until now, there is no strong evidence showing the superiority of using one non-biological immunosuppressive agent over the other [4].

In this case, the patient showed good clinical improvement upon administration of steroids and methotrexate; nevertheless, there was no laboratorial or radiological improvement. After reduction of steroids, the patient relapsed showing new symptoms, and cyclophosphamide was added to the first regimen. Upon adding cyclophosphamide, the patient showed no clinical or laboratorial improvement during the course of 8 months. Glucocorticoids and cyclophosphamide were continued, and methotrexate was replaced with azathioprine. Finally, this led to the remission of both clinical symptoms and acute phase reactants without radiological improvement, and the patient was continued on steroids and azathioprine.

Supportive therapy includes the use of antiplatelets and antihypertensive agents in patients suffering from hypertension [6].

As for surgery, which plays an important role as an essential treatment in some cases, especially in medically unresponsive progressive TA [9], our patient did not match any indication for surgical treatment.

## Conclusion

Childhood Takayasu arteritis is a life-threatening disease that can have varied presentation. There is a scarcity of reviews and studies on c-TA in Arabs. As mentioned earlier, there is a low prevalence of c-TA in the Arab population. However, lack of awareness toward the disease among primary care physicians may be a reason for its low prevalence. The main purpose of our report is to share our experience and to keep TA in mind as a differential diagnosis in any child presenting with hypertension, absent or reduced peripheral arterial pulse, or blood pressure difference between arms.

## Data Availability

Any additional data or material is available on request.

## Abbreviations

WBCs: White blood cells
CRP: C-reactive protein
ESR: Erythrocyte sedimentation rate
TA: Takayasu arteritis
c-TA: Childhood Takayasu arteritis
BP: Blood pressure
MRA: Magnetic resonance angiography
